# Coronary heart disease prediction method fusing domain-adaptive transfer learning with graph convolutional networks (GCN)

**DOI:** 10.1038/s41598-023-33124-z

**Published:** 2023-08-31

**Authors:** Huizhong Lin, Kaizhi Chen, Yutao Xue, Shangping Zhong, Lianglong Chen, Mingfang Ye

**Affiliations:** 1 Department of Cardiology, Fujian Heart Medical Center, Fujian Institute of Coronary Heart Disease, Fujian Medical University Union Hospital, Fuzhou, 350001 People’s Republic of China; 2 College of Computer and Data Science, Fuzhou University, Fujian, 350108 People’s Republic of China

**Keywords:** Cardiovascular diseases, Computer science

## Abstract

Graph convolutional networks (GCNs) have achieved impressive results in many medical scenarios involving graph node classification tasks. However, there are difficulties in transfer learning for graph representation learning and graph network models. Most GNNs work only in a single domain and cannot transfer the learned knowledge to other domains. Coronary Heart Disease (CHD) is a high-mortality disease, and there are non-public and significant differences in CHD datasets for current research, which makes it difficult to perform unified transfer learning. Therefore, in this paper, we propose a novel adversarial domain-adaptive multichannel graph convolutional network (DAMGCN) that can perform graph transfer learning on cross-domain tasks to achieve cross-domain medical knowledge transfer on different CHD datasets. First, we use a two-channel GCN model for feature aggregation using local consistency and global consistency. Then, a uniform node representation is generated for different graphs using an attention mechanism. Finally, we provide a domain adversarial module to decrease the discrepancies between the source and target domain classifiers and optimize the three loss functions in order to accomplish source and target domain knowledge transfer. The experimental findings demonstrate that our model performs best on three CHD datasets, and its performance is greatly enhanced by graph transfer learning.

Coronary heart disease (CHD) is a serious cardiovascular disease (CVD)^1^ that not only has a high mortality rate but also patients are prone to the risk of recurrence even after they are cured and discharged from the hospital, both of which are prognosis-oriented factors for poor outcomes. The root cause of coronary heart disease is the persistent buildup of fat or unhealthy cholesterol inside the artery wall, which eventually causes the artery wall to narrow and block^2^. Arrhythmia, myocardial infarction, and angina pectoris symptoms are the most common clinical signs of coronary heart disease. The main methods currently used in clinical practice for the detection of coronary artery disease include^3^: electrocardiogram (ECG), ECG stress test, echocardiography, ambulatory ECG, hematology, CT angiography, and other techniques. Among these examination methods, the diagnosis is usually made by a cardiologist by examining the ECG waveform, relying on the expert's long-term experience and subjective judgment. But these decisions can be supported and replaced to some extent by automated data-driven methods.

Deep learning (DL) techniques overcome the limitations of medical computer-aided diagnosis (CAD) systems to automatically extract discriminative features on image data^4^. End-to-end learning systems involving risk assessment prediction for diseases have four main steps, such as feature extraction, segmentation, normalization, and smoothing. Convolutional neural networks (CNNs) provide the best solution for classification in many fields, such as computer vision (CV), medical imaging, and natural language processing (NLP)^5^. Graph embedding algorithms mainly focus on the neighborhood of nodes and edges to extract knowledge, such as Netpro2vec^6^. However, deep learning approaches for healthcare have some limitations when training models using specific data, as the model requires large amounts of annotated medical data (e.g., images, text). The availability of large amounts of annotated data is complicated by privacy and cost issues.

Healthcare systems produce enormous volumes of raw data every day in the big data age. Manual labeling is costly, data is easily outdated, and access to labeled data is difficult. In this instance, it's possible that the labeled data acquired during one time period won't have a similar distribution throughout the subsequent time period. For cardiovascular data, data from patient clinical visits reflects postoperative recovery, while abnormal data often arises from rhythm variability at future moments^7^. Therefore, medical systems have to continuously collect immediate data like ECG. For deep learning, such datasets are always changing, the cost of training is increasing, and the difficulty of migration is also prominent. And transfer learning can handle these needs effectively.

Recently, transfer learning (TL) has been used in the fields of medical imaging, medical healthcare, etc. Transfer learning^8,9^ seeks to take information from one or more source tasks and apply it to the target task. Xu et al.^10^ proposed a deep-transfer CNN framework for EEG classification. It transfers the pre-trained VGG-16 CNN model to the target CNN model for EEG classification, and improves its classification accuracy through fine-tuning of post-layer parameters. However, the transfer ability of the pre-trained CNN model on different layers has not been investigated. Tadesse et al.^11^ proposed an end-to-end trainable cross-domain transfer learning CVD diagnostic model that analyzes the fusion of multiple ECG leads in combination with spectrogram stacking alignment. However, the high-dimensional processing of CNN features was neglected. Lakshmi et al.^12^ used CNN architecture and transfer learning techniques to measure carotid intima-media thickness (CIMT) from ultrasound images of type II diabetic subjects in combination with the Framingham Risk Score (FRS) to achieve a good prediction of CVD risk. However, it is difficult to transfer to other cardiovascular datasets.

However, research on transfer learning has mainly focused on the fields of CV and NLP. Images in CV and sequence samples in NLP are independently distributed, and they rarely require rotation invariance of the model. Unlike CNN, such network-structured data with nodes and edges requires model rotation invariance. For example, in biochemistry, isomorphic graphs of drug molecules exhibit different effects^13^. Therefore, TL cannot be directly applied to problems such as node classification, link prediction, and graph classification in graph neural networks (GNN)^14–18^. It is due to the phenomenon of graph isomorphism that such network-structured data with nodes and links with different relational edges requires rotation invariant models. When models need to be transmitted across networks to handle the same task, they may suffer from embedding space drift^19^ and differences in embedding distributions^20^. Existing methods rarely model the GNN cross-domain network structure information, which is central to node classification.

In summary, the following challenging problems are still faced in CHD risk classification applications: (1) Deep learning models often need to be trained on a lot of labeled data. However, most CHD datasets are unlabeled or missing, and experts cannot manually label all samples. (2) Training deep learning models from scratch is very time-consuming and computationally intensive. (3) There may be significant differences between source and target data across domains, with few common attributes. To address these three challenges, in this paper, we propose a supervised adversarial domain-adapted multichannel graph convolutional network (DAMGCN) for cross-domain node classification. It models the local and global consistency relationships of each graph and combines source information, adversarial domain, and target information into a unified deep model. The main contributions and innovations of the article are as follows:

- We study the graph transfer learning problem on the CHD dataset for the first time , where the already labeled source network is used to assist the target network for node classification by means of graph convolution and adversarial domain adaptation.
- In the node feature extraction layer, we use the local and global consistency relations of each graph to assist the training of the node embedding module. And the attention mechanism is used to unite the local and global relations for a comprehensive node representation of each domain.
- We consider source domain information, adversarial domain information and target domain information simultaneously, and learn domain invariant and semantic representations to reduce domain differences across domains. Cross-domain experiments on three real CHD datasets show that DAMGCN outperforms state-of-the-art single-domain graph representation learning models.

## Results

### Dataset description

The cardiovascular medicine division of a tertiary care hospital in the Fujian Province of China contributed some of the genuine hospital patient data that was used in this investigation. The dataset includes data from CHD patients collected through return visits during the five-year period from 2016 to 2021. It includes 5850 patients who had procedures (coronary angiography and PCI for revascularization) discharged from the hospital, with 430 records for each index per patient, totaling approximately 2515,500 records. The original dataset contains a large number of missing patient records, as well as problems such as noise and irregularities. We did data preprocessing, including data cleaning, data conversion, missing value filling, and redundant data removal operations. Finally, the dataset was divided into seven major categories: basic patient information; past medical history; ECG indexes; cardiac ultrasound indexes; phlebotomy indexes; medication consumption; and coronary vascular lesions^21^.

### Ethics approval

This research was approved by the Institutional Review Board (IRB) of Fujian Medical University Union Hospital (Approval number: 2021KJCH082). Interviews were performed after confirmation of informed consent. confirming that informed consent has been obtained from all subjects and their legal guardians. This consent process was approved by the ethics committee. All experiments were performed in accordance with relevant guidelines and regulations. H.Z.L. had access to information that could identify individual participants during or after data collection.

We re-extracted two datasets related to all-cause mortality from the original dataset. We constructed graphs for both datasets based on the KNN approach^22^. The details of the experimental datasets are shown in Table 1. All-cause death, Heart level, and Mace occurrence are CHD datasets derived from three different branches under the Cardiovascular Division. For each dataset, we extracted a subset and a number of important features that were relevant to the source task, and the segmentation targets were ultimately highly correlated with patient deaths. In our experiments, we treat them as undirected networks, with each edge representing a similar relationship between patients. We classified each dataset into two categories based on patient outcomes, including "Death (yes)", "Death (no)", "Cardiac function (high risk)", "Cardiac function (low risk)", "Mace (occurred)" and "Mace (did not occur)". We investigated this through six transfer learning tasks, including H → D, M → D, D → H, M → H, D → M, and H → M, where D, H, and M denote all-cause death, heart level, and Mace occurrence, respectively.

**Table 1:** Statistics of the CHD dataset in the experiment.

Dataset	Abbreviation	# of Nodes	# of Edges	# of Features	# of Labels
All-cause death	D	2702	9383	25	2
Heart level	H	1345	3895	25	2
Mace occurrence	M	1210	4046	25	2

### Baseline

To make a fair comparison with the model proposed in this paper, we use the following four methods as a baseline. Specifically, we compare this paper's approach with an advanced single-domain node classification model to verify the superiority of the cross-domain model. Later, the necessary comparisons are made between the variants of the model in this paper to analyze the role of each component.

1. DNN**:** DNN is a multilayer perceptron (MLP) that uses only node features.
2. GCN: GCN^16^ is a great deep convolutional network for graph-structured data that incorporates network topology and node attributes into a comprehensive learning framework. By default, we use KNN as the composition strategy (denoted as k-GCN).
3. p-GCN: A type of GCN, the graph structure used by the model is in the form of a population graph^18^ as the main approach.
4. AM-GCN: The optimal single-domain graph convolution model studied in ^18^ is characterized by having adaptive dual-channel graph convolution layers and attention layers represented as aggregated nodes to accommodate node classification for different tasks.
5. DAMGCN: The model proposed in this paper. It is capable of performing graph convolution models for cross-domain node classification.

### Experimental parameters

The experimental language uses Python 3.8.11, Scikit-learn 0.24.2, and the environment is the Pytorch 1.6.0 framework. Table 2 gives the experimental parameter settings. Our experimental parameters are carried out according to a uniform standard. We employ the Adam optimizer for training and a fixed learning rate of $3e-3$ for all deep model approaches. We use all labeled source data as well as target data with a divided training (60%) and testing (40%) ratio. We apply the same set of parameter configurations to all cross-domain node classification tasks. For GCN, AMGCN, and DAGCN, the GCNs of both the source and target networks contain two hidden layers with a structure of 128–16. The dropout rate of each GCN layer is set to 0.5. The DNN has similar parameter settings as the GCN. Thus, the adaptation rate λ is calculated as follows: $\lambda =min\left(\frac{1}{1+exp\left(-10p\right)}-\mathrm{1,0.1}\right)$, where p changes from 0 to 1 during the training process.

**Table 2:** Parameter configuration of model.

Parameter name	Parameter value	Parameter description
Epochs	300	Training batch size
k	6	Number of edges (KNN)
lr	0.003	Learning rate
nhid1	128	Number of hidden layers 1
nhid2	16	Number of hidden layers 2
Dropout	0.5	Drop rate
γ1	1	Balance parameter 1
γ2	0.8	Balance parameter 2
Seed	21	Random number seed

### Performance indicators

In this work, we performed the task related to dichotomous classification on three CHD datasets. The results of the experiment were evaluated specifically using Accuracy, Precision, Recall, F score, and AUC (area under the ROC curve)^23^. The dataset is imbalanced, and we also use MCC and balanced accuracy metrics to reflect its performance^24^. True positive (TP) and true negative (TN) values indicated correctly classified malignant and benign cases, respectively. False-positive (FP) and false-negative (FN) values indicate incorrectly classified benign and malignant cases, respectively. In our experiments, we used the macro-averaging technique to calculate the Precision, Recall, and F score of the model.

### Model comparison results

We applied the advanced single domain model on 3 CHD datasets with the results shown in Table 3. The DNN, p-GCN, k-GCN, and AMGCN are the experimental results from previous work^18^. Their respective advantages and limitations are summarized in Table 4. We mainly focus on the D (all-cause death) risk classification task for detailed analysis. Among these baselines, DNN has the worst performance (44.6% MCC, 72.2% F1 score). This is due to the fact that typical DNNs only take into account node attributes and do not collect information about the network structure to better represent nodes; graph-based approaches (GCNs) have better performance than traditional approaches (63.2–63.5% MCC, 71.5–73.4% balanced accuracy), which suggests that graph convolutional neural networks encoding local graph structure and node features have a competitive advantage over traditional models for node classification. The performance of AMGCN is the best in single-domain node classification, having 97.3% Accuracy, 90.4% AUC, and 80.9% F1-score (0.6%, 3.7%, and 5.3% higher for DAMGCN on H- > D tasks, respectively). However, AMGCN needs to be retrained in each single-domain classification, which takes a long time and cannot perform task transfer. The DAMGCN model proposed in this paper consistently outperforms all single-domain classification methods on cross-domain tasks (74.6% MCC, 81.1% balanced accuracy), demonstrating the advantage of adversarial domain adaptation in the classification of cross-domain nodes.

**Table 3:** Predicted results of all-cause death (indexes 7–8 are predictions of heart level, 9–10 are predictions of Mace occurrences).

Index	Model	Accuracy (%)	AUC (%)	Precision (%)	Recall (%)	F1score (%)	MCC (%)	Balanced accuracy (%)
1	DNN	95.4	78.7	74.2	70.5	72.2	44.6	70.5
2	p-GCN	96.6	87.7	86.1	70.2	75.7	52.4	69.3
3	k-GCN	97.2	88.6	96.5	71.5	79.0	63.2	71.5
4	AM-GCN	97.3	90.4	93.4	74.4	80.9	63.5	73.4
5	DAMGCN(H)	97.9	94.1	93.5	81.2	86.2	74.6	79.4
6	DAMGCN(M)	97.9	95.1	95.9	79.3	85.6	71.6	81.1
7	DNN	85.1	77.4	76.4	74.0	75.0	50.3	74.0
8	GCN	89.4	86.1	79.6	89.0	75.2	62.7	75.2
9	DNN	92.1	65.4	66.2	62.4	64.0	28.4	62.4
10	GCN	91.8	88.7	86.2	93.6	82.1	62.4	71.8

**Table 4:** Summary of previous models.

References	Method	Advantage	Limitations
^18^	DNN	Better performance than traditional methods (SVM, GBDT, etc.)	1). Inability to transfer learning tasks; 2). Unable to get graph node representation;
^16,22^	GCN, KNN	Node representation embedding; Simple construction graphs	1). Inability to transfer learning tasks; 2). Highly affected by the graph structure (k value);
^18^	GCN, Population Graph	Node representation embedding; Suitable for medical scenarios	1). Inability to transfer learning tasks; 2) Slow construction of graphs;
^18^	AMGCN	Multi-channel information aggregation; attention mechanism	1). Inability to transfer learning tasks; 2). Long model training time; 3). High calculation volume;
[–]	DAMGCN	Cross-domain transfer learning; global consistency	1). Unable to train on a single domain 2). requires the source domain to be relevant

### Cross-domain classification results

By comparing the cross-domain node classification, we observed an improvement in the predictive performance of different domains for All-cause death. In particular, although single domains can distinguish between true-negative and true-positive patient cases to some extent, there is an unbalanced distribution of sample proportions and an underdetection of death classes, which can be reflected in MCC (63.5%) and balanced accuracy (73.4%) performance. And after adversarial domain transfer, this problem can be improved. The best AUC performance of the model was achieved for the transfer of Heart level (H) to All-cause death (D). The AUC was improved by 4.7%, Recall by 4.9%, and F1 score by 4.7%. Thus, groups with a high Heart level contribute to the screening of All-cause death groups. For Mace occurrence (M) transferred to All-cause death(D), the best positive case prediction for All-cause death was achieved. The AUC was boosted by 3.7%, Recall by 6.8%, and F1 score by 5.3%. Thus, Mace occurrence and All-cause death data were similarly distributed and highly correlated, and both were highly transferable.

Table 5 shows the performance of the proposed model in different cross-domain node classification tasks. The findings lead to the following conclusions: (1) Compared with the single-domain prediction results in Table 3, it is clear that the cross-domain model proposed in this paper has better prediction results for specific CHD tasks (all-cause death, cardiac function, and Mace occurrence). Overall, the performance of each metric is improved; (2) different source domains have different effects on the target domain migration. For the D dataset, the knowledge provided by H was more helpful for the Recall metric, while M corresponded to the AUC metric. For the H dataset, the knowledge provided by D is more helpful for AUC and Recall metrics, while M corresponds to Precision and Accuracy. For the M dataset, the knowledge provided by D is more helpful for F1 score and Recall metrics, while H corresponds to Precision; (3) Since adversarial domain adaptation in model training tries to find an optimal solution method, both continuously reducing the source and target classifier differences and keeping each classifier trained optimally on its respective target task, it is also improving the source domain performance when improving the target domain.

**Table 5:** Comparison of classification performance for six cross-domain tasks.

Source	Target	Accuracy (%)	AUC (%)	Precision (%)	Recall (%)	F1score (%)	MCC (%)	Balanced accuracy (%)
H	D	97.9	94.1	93.5	81.2	86.2	74.6	79.4
M	D	97.9	95.1	95.9	79.3	85.6	71.6	81.1
D	H	94.8	96.5	93.4	89.5	91.3	81.4	87.0
M	H	95.0	96.3	94.7	88.8	91.4	84.0	88.2
D	M	96.9	95.7	93.8	79.5	85.0	71.7	76.6
H	M	96.7	95.7	95.6	76.5	83.1	67.2	73.4

The results show that by jointly modeling the consistency relationships of each graph, domain information, source domain information, and target domain information into a unified learning framework, the proposed DAMGCN can better capture the underlying representation of graph nodes and reduce the distribution gap across domains.

Table 6 summarizes the graph neural network models used in the paper and their different variants, where the symbolic √ representation algorithm utilizes the corresponding information. The specific analysis will be presented in the next part.

**Table 6:** Summary of each graph convolutional network.

Model	Local	Global	Attention	Domain loss	Target loss
GCN	√				
AMGCN	√		√		
DAMGCN¬t	√	√	√	√	
DAMGCN¬g	√			√	√
DAMGCN¬d	√	√	√		√
DAMGCN	√	√	√	√	√

The symbol √ denotes that the associated information is used by the algorithm.

### Analysis of variant components

Since the proposed DAMGCN contains several key components, we compare variants of DAMGCN in this section to demonstrate the superiority of DAMGCN in terms of (1) the effect of target classifier loss; (2) the effect of the global GCN layer module; and (3) the effect of domain adversarial loss. The details are described as follows.

1. **DAMGCN¬t**. To demonstrate the effectiveness of cross-domain transfer learning, we designed a variant model, DAMGCN¬t, to simulate the case of direct parameter migration. The only difference between DAMGCN¬t and DAMGCN is that DAMGCN¬t removes the target classifier loss from the model, i.e., it does not use information from the target domain, which is the core of cross-domain learning . The results in Table 7 show that when the target classifier loss is not used, the migration effect for all-cause death decreases by 15.07% (H- > D) and 6.94% (M- > D), and the rest of the cross-domain tasks also have substantial decreases. This indicates that the migration effect brought about by directly using model parameters and domain adversarial variance reduction is not good. This would allow the target classifier to gradually approximate the source classifier when, in fact, the target domain information may be very different from the source domain.

**Table 7:** Comparison of the classification accuracy of DAMGCN variants on six cross-domain tasks.

Methods	H → D	M → D	D → H	M → H	D → M	H → M
DAMGCN¬t	0.7280	0.9093	0.7472	0.8197	0.9194	0.8326
DAMGCN¬g	0.9704	0.9732	0.8941	0.8903	0.9463	0.9463
DAMGCN¬d	0.9695	0.9732	0.8885	0.9015	0.9442	0.9504
DAMGCN	0.9787	0.9787	0.9480	0.9498	0.9690	0.9669
2. **DAMGCN¬g**. It belongs to a variant of DAMGCN that removes the global GCN layer of DAMGNN and uses only the local GCN layer. The effectiveness of the global GCN approach is investigated by comparing the two. The key between them is the presence or absence of using the PMI matrix. From the results, we found that the DAMGCN model outperformed DAMGCN**¬**g, with an improvement of 0.83% (H- > D) and 0.55% (M- > D) for all-cause death migration. Similarly, there was some degree of accuracy improvement for both the cross-domain tasks at the heart level and Mace occurrence. This confirms that the ability to extract node information using only the local GCN layer is limited, while the addition of the global GCN layer module containing the PMI matrix enables us to uncover potential relational nodes. And it combines local and global relations to capture a comprehensive representation of nodes.
3. **DAMGCN¬d**. To verify the effectiveness of domain adversarial loss, we compared the DAMGCN model with DAMGCN¬d. DAMGCN¬d removes the gradient inverse layer of DAMGCN, i.e., the domain classifier. Without using domain adversarial loss, the source and target domains each train their own models and share some parameters, and this knowledge transfer is weak. Figure 1 shows the DAMGCN variant for different cross-domain task accuracies. We can easily observe the superiority of the DAMGCN model with a migration improvement of 0.92% (H- > D) and 0.55% (M- > D) for all-cause death. Similarly, some degree of accuracy improvement was observed for both heart level and Mace occurrence for the cross-domain task. On the one hand, this confirms that better node representations can be learned from different domains using adversarial domain loss. On the other hand, adversarial domain loss does help to reduce the difference between two different domains during model training, improving the training effect and migrability to the target domain.

**Figure 1:**
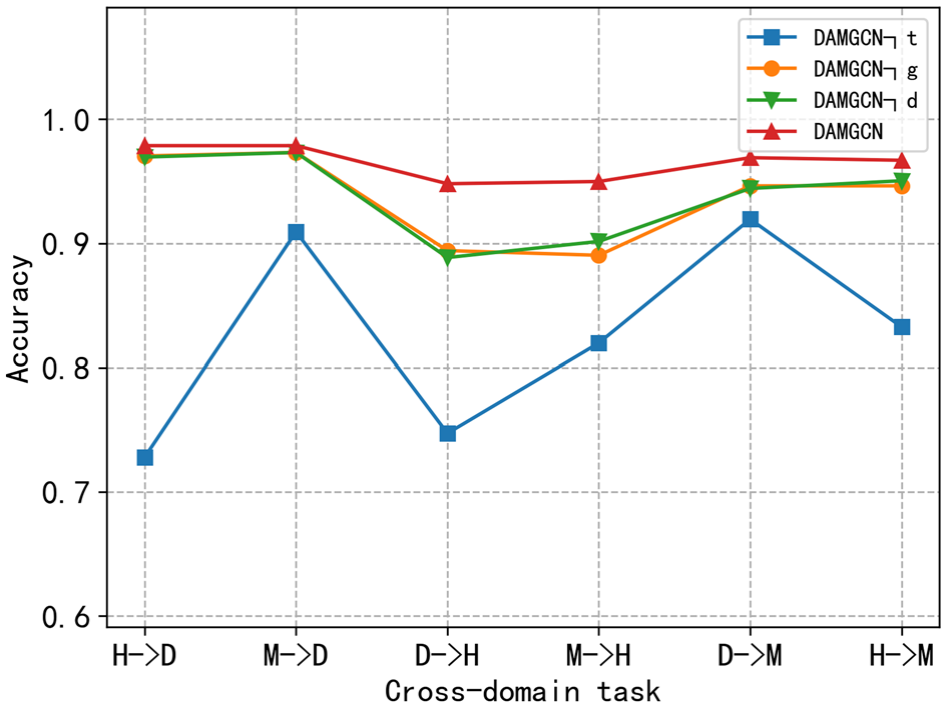
Accuracy performance of DAMGCN and its variants on different cross-domain tasks.

### Visualization

**Figure 2:**
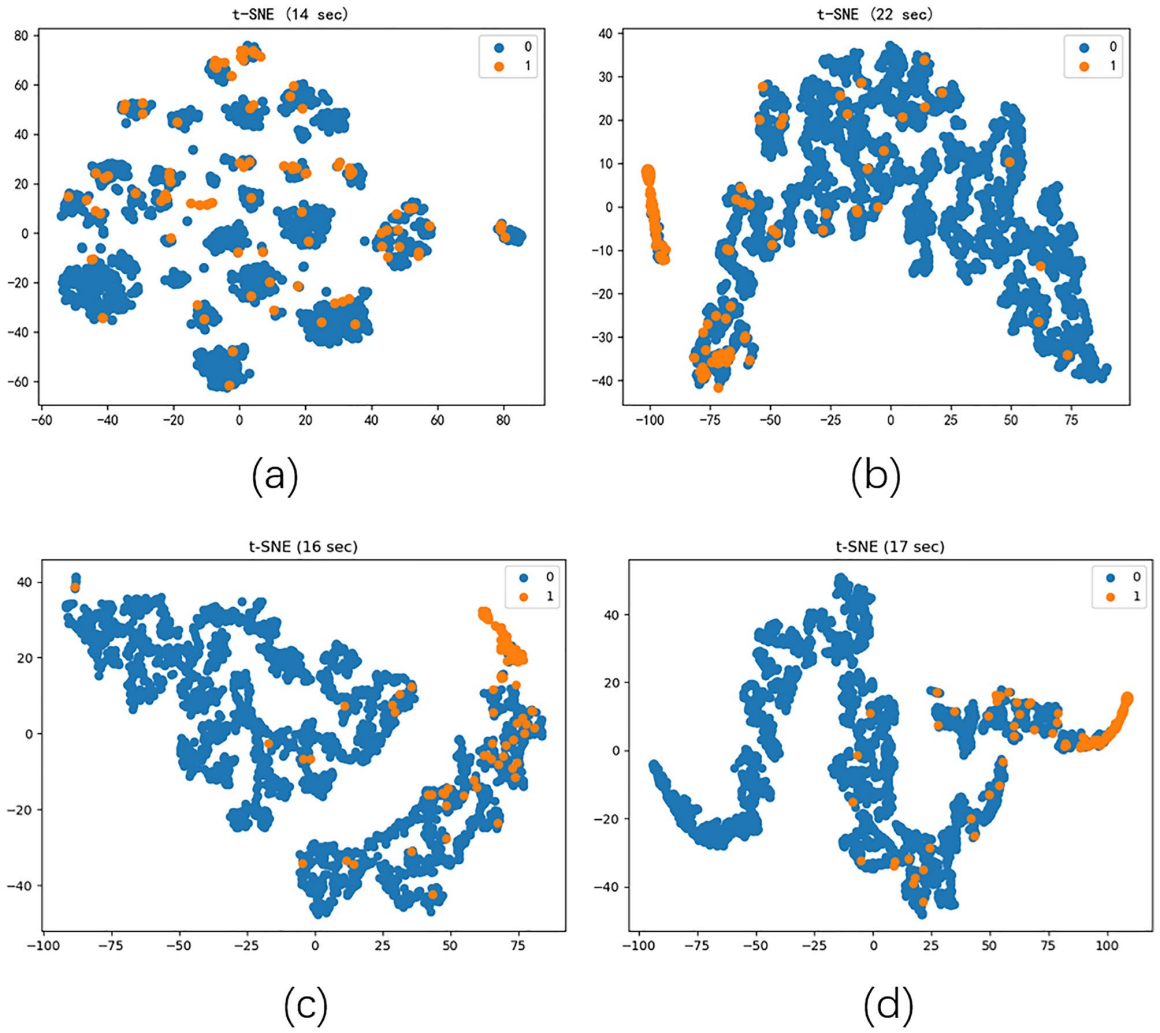
Visualization of D dataset graph embedding learning results using t-SNE (**a** is the original embedding, **b** is the GCN, **c** is the DAMGCN for H- > D, and **d** is the DAMGCN for M- > D).

**Figure 3:**
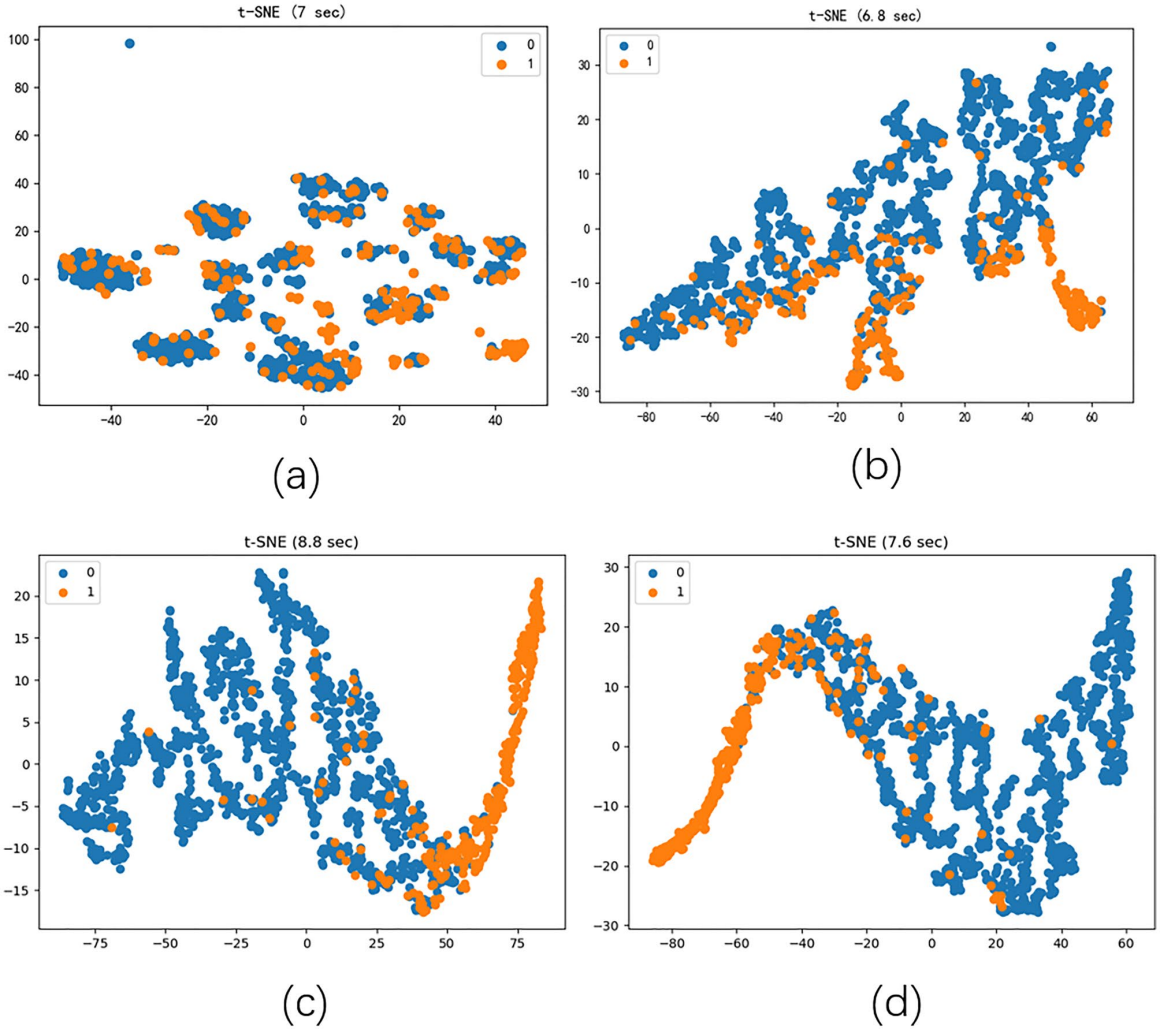
Visualization of embedding learning results for the Figure H dataset using t-SNE (**a** is the original embedding, **b** is the GCN, **c** is the DAMGCN for D- > H, and **d** is the DAMGCN for M- > H).

**Figure 4:**
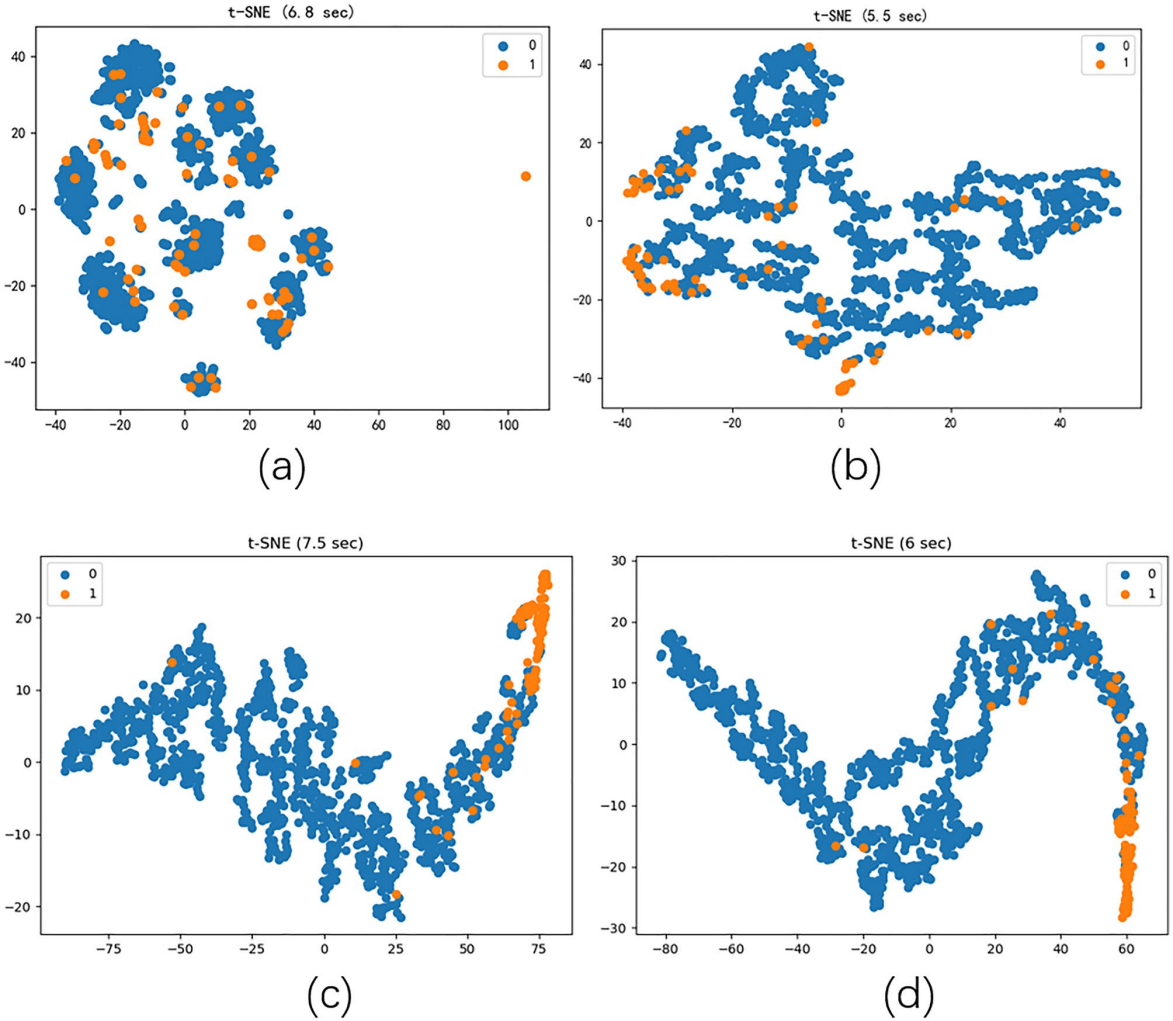
Visualization of M dataset graph embedding learning results using t-SNE (**a** is the original embedding, **b** is the GCN, **c** is the D- > M DAMGCN, and **d** is the H- > M DAMGCN).

To demonstrate the effectiveness of our proposed model more visually, we performed the visualization task on three CHD datasets. We used the t-SNE^25^ method to map the learned embedding vectors into a two-dimensional space before the original features and the last layer of the depth model for each task, respectively. The presentation results of the datasets in Figs. 2, 3, and 4 are colored by the real labels. Observing each figure, we can find that the original feature distribution is chaotic and it is difficult to distinguish between positive and negative class nodes; although GCN can distinguish the respective regions of the classes, there is also a mix of nodes with different labels. Obviously, DAMGCM has the best visualization effect. It learns a more compact embedding structure with the highest inter-class similarity and clear boundaries between different classes.

## Methods

**Figure 5:**
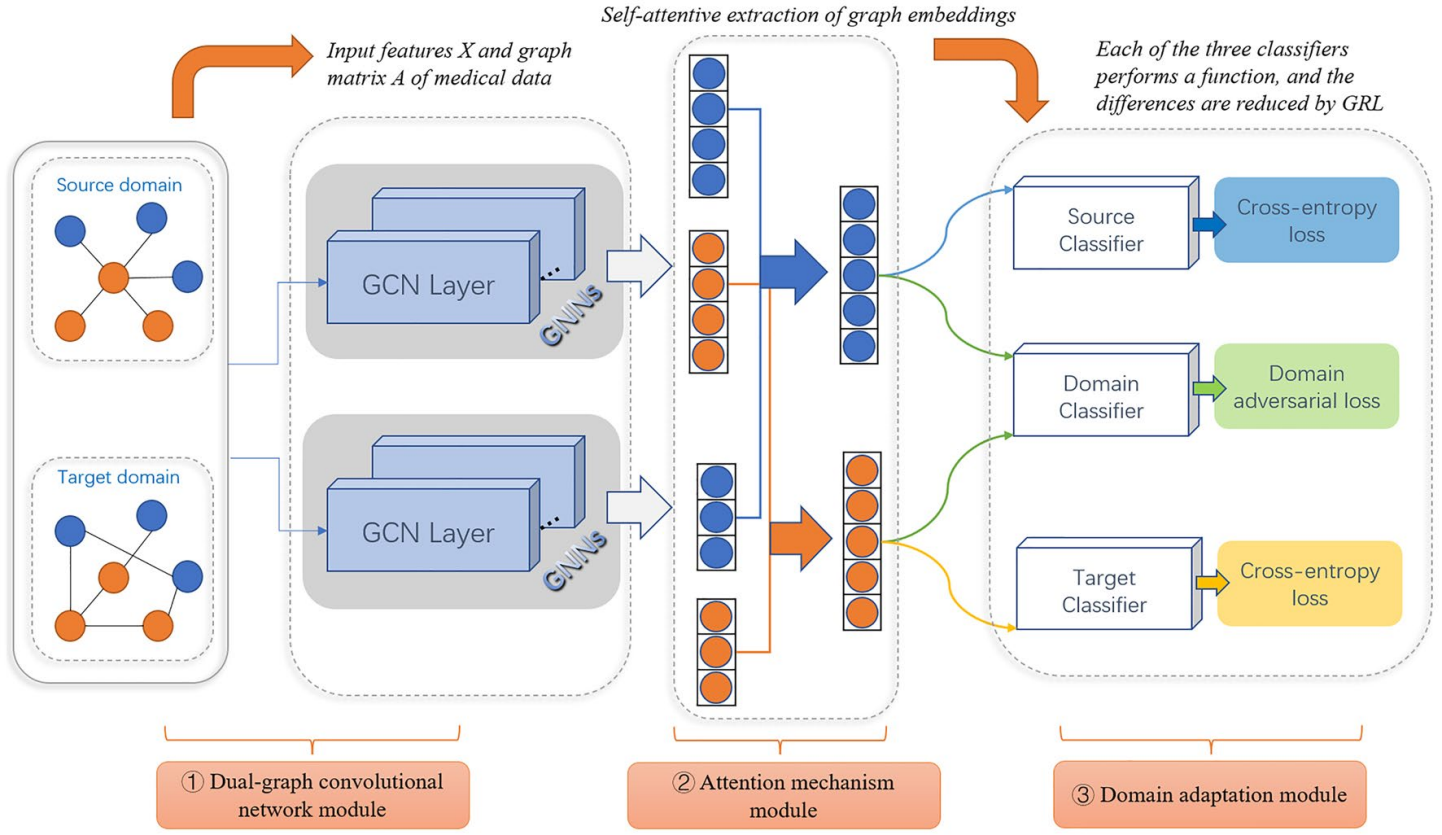
Overall architecture of the proposed adversarial domain-adapted multichannel graph convolutional network (DAMGCN) for cross-domain node classification.

All the methods under this study were performed in accordance with the relevant guidelines and regulations of Fuzhou University (Fzu). The experimental protocols were approved by the Institutional Review Board (IRB) of Fujian Medical University Union Hospital. Informed consent was taken from all participants. In this section, we present our domain-adaptive graph convolutional network for cross-domain node classification. It is improved by the unsupervised domain adaptive network model proposed by Wu^26^. Our model consists mainly of three parts: (1) a dual-graph convolutional network module; (2) an attention mechanism module; and (3) a domain adaptation module. The DAMGCN model is shown in Fig. 5.

### The proposed model (DAMGCN)

Given a source domain ${G}^{S}=\left({V}^{S},{E}^{S},{X}^{S},{Y}^{S}\right)$ and a target domain ${G}^{T}=\left({V}^{T},{E}^{T},{X}^{T},{Y}^{T}\right)$, the goal of our model is to obtain node representations for the source domain graph embedding ${Z}^{S}$ and the target domain graph embedding ${Z}^{T}$ through supervised training. The training process continuously narrows the domain differences, shares the training parameters from the source domain to the target domain, and improves the classification ability of similar tasks in the target domain. First, we use a dual graph convolutional network structure to capture the local and global consistency relations of each graph separately. Among them, the initial inputs to the source and target domains are ${X}^{S}$ and ${X}^{T}$, and the outputs are ${Z}_{A}^{S},{Z}_{P}^{S},{Z}_{A}^{T}$, and ${Z}_{P}^{T}$. Then, we apply the graph attention mechanism to the output of each domain to obtain the final outputs of node representations ${Z}^{S}$ and ${Z}^{T}$. Finally, we can effectively learn domain invariant and semantic representations to eliminate domain disparities in cross-domain node classification by employing the source classifier, domain adversary, and target classifier.

### Deep transfer learning

**Figure 6:**
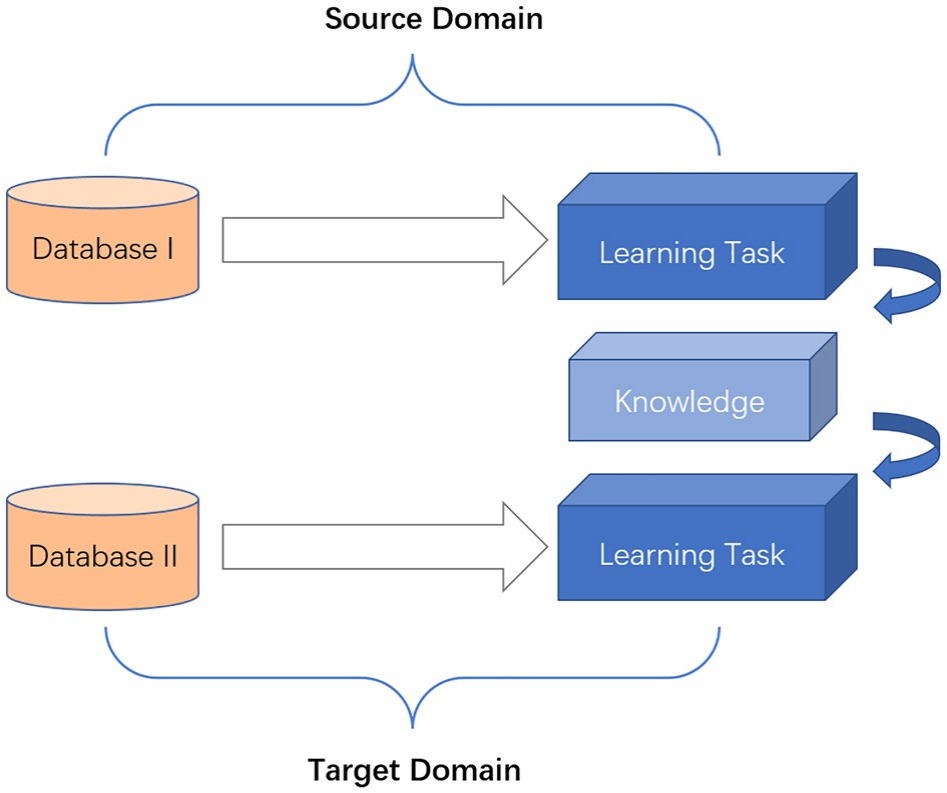
General deep transfer learning process.

The important mathematical notation in deep transfer learning^9^ is defined as follows: the domain can be represented as $D=\left\{X,P\left(X\right)\right\}$, where $\left\{{x}_{i},\dots ,{x}_{n}\right\}\in X$ is the feature space and $P\left(X\right)$ is the edge probability distribution. A task can be represented by $T=\left\{ y,f\left(x\right)\right\}$. It consists of two parts: the label space $y$ and the target prediction function $f\left(x\right)$. $f\left(x\right)$ can also be viewed as the conditional probability function $P\left(y | x\right)$. The deep migration task can be defined as $\left\{{D}_{s},{D}_{t}, {T}_{s},{T}_{t},{f}_{t}\left(\cdot \right)\right\}$. Given a learning task ${T}_{t}$ based on ${D}_{t}$. Deep transfer learning aims to discover and transfer potential common knowledge from ${D}_{t}$ to ${T}_{t}$ and improve the performance of the prediction function ${f}_{t}\left(\cdot \right)$ on the learning task ${T}_{t}$. where it is sufficient to satisfy ${D}_{s}\ne {D}_{t}$ or ${T}_{s}\ne {T}_{t}$. The generic transfer learning process is shown in Fig. 6.

### Deep domain adaptation

Domain adaptation is a sub-theme of transfer learning that aims to migrate from a source domain with sufficient labeling information to a target domain with a large amount of unlabeled or little labeled data by minimizing domain differences^27–29^. Domain adaptation can mitigate the detrimental effects of domain drift that occurs when knowledge is migrated from a source to a target by facilitating migration with models in different but related domains that share the same label space^30,31^. Several studies have also attempted to apply domain adaptation ideas to graph-structured data. The CDNE^32^ algorithm learns transferable node embeddings for cross-network learning tasks by minimizing the maximum mean difference (MMD) loss. But its modeling capability is limited. The AdaGCN algorithm^33^ uses graph convolutional networks as feature extractors to learn node representations and uses adversarial learning strategies to learn domain-invariant node representations. But it ignores the semantic information contained in the target domain samples. The UDA-GCN algorithm^26^ works similarly to ours, using a composite framework in order to achieve knowledge adaption between graphs. But it studies the problem of graph domain adaptation in unsupervised scenarios.

### Point-by-point mutual information matrix

Mutual information measures the amount of information contained in a given random variable about another random variable. It involves the reduction of uncertainty in one random variable due to the presence of another random variable. Assuming that the two random variables are independent, point-by-point mutual information (PMI) measures the difference between the co-occurrence probability of the marginal distribution given X and Y and the co-occurrence probability given their joint distribution^34^. The PMI metric performs well in semantic similarity tasks.

In order to calculate the PMI matrix (denoted as P), we first use the random walk-based method to calculate the frequency matrix. Random wandering has been used as a similarity metric in semi-supervised graph representation learning and graph classification problems^35^. The random walk utilizes the entire graph information to calculate the neighbor probability of each node. The semantic similarity between different nodes can be calculated by the random walk method. Then, using the semantic knowledge from frequency to frequency-based matrix, we finally get the PMI matrix.

1. **Frequency matrix:** A random wander is a Markov chain that describes the order of all nodes visited throughout the wander. If the random wanderer is on node ${x}_{i}$ at time $t-1$ per unit time, the state at this point is defined as $s\left(t-1\right)={x}_{i}$. If the current node ${x}_{i}$ jumps to any neighbor ${x}_{j}$ at the next moment $t$, the transfer probability is $p\left(s\left(t\right)={x}_{j}|s\left(t-1\right)={x}_{i}\right)$. Given an arbitrary adjacency matrix ${A}_{i,j}$, the transition probabilities of all nodes in A are as follows:
  1 $$\begin{array}{c}p\left( s\left(t\right)={x}_{j}|s\left(t-1\right)={x}_{i} \right)=\frac{{A}_{i,j}}{\sum_{j}{A}_{i,j}}\end{array}$$
2. **P matrix:** From the frequency matrix (denoted F) obtained from Eq. (1), we define the context of the input as all nodes in X. The visited path is equivalent to a sentence, and the node corresponds to a word. Its i-th row is the row vector ${F}_{i,:}$ and the j-th column is the column vector ${F}_{:,j}$. ${F}_{i,:}$ corresponds to node ${x}_{i}$, and ${F}_{:,j}$ corresponds to the context ${c}_{j}$. The number of occurrences of ${x}_{i}$ in the context of ${c}_{j}$ is the value of the entry ${F}_{i,j}$. Based on the frequency matrix F, we compute the P by the following equation:
  2 $$\begin{array}{c}{P}_{i,j}=\frac{{F}_{i,j}}{\sum_{i,j}{F}_{i,j}}\end{array}$$
  3 $$\begin{array}{c}{P}_{i,*}=\frac{\sum_{j}{F}_{i,j}}{\sum_{i,j}{F}_{i,j}}\end{array}$$
  4 $$\begin{array}{c}{P}_{*,j}=\frac{\sum_{i}{F}_{i,j}}{\sum_{i,j}{F}_{i,j}}\end{array}$$
  5 $$\begin{array}{c}{P}_{i,j}=max\left\{\mathrm{log}\left(\frac{{P}_{i,j}}{{P}_{i,*}{P}_{*,j}}\right), 0\right\}\end{array}$$

According to Eqs. (2) to (5), the semantic information in P is encoded. ${P}_{i,j}$ represents the estimated probability of node ${x}_{i}$ occurring in context ${c}_{j}$; ${P}_{i,*}$ represents the estimated probability of node ${x}_{i}$; and ${P}_{*,j}$ represents the estimated probability of context ${c}_{j}$. According to the definition of statistical independence, if ${x}_{i}$ is independent and occurs randomly with respect to ${c}_{j}$, then ${P}_{i,j}={P}_{i,*}{P}_{*,j}$, so ${PMI}_{i,j}=0$. If ${x}_{i}$ and ${c}_{j}$ have a semantic relationship, then ${P}_{i,j}>{P}_{i,*}{P}_{*,j}$ and ${PMI}_{i,j}$ have positive values. Similarly, if ${x}_{i}$ is uncorrelated with ${c}_{j}$, ${PMI}_{i,j}$ may be negative. Since we are interested in pairs $\left({x}_{i},{c}_{j}\right)$ with semantic relations, our approach uses non-negative ${PMI}_{i,j}$.

### Global consistency

In^36^, it demonstrates that the graph's global information is more important. Our model trains two GNN modalities to extract features, thereby capturing both local and global information about the graph network. And we encode the semantic information of each node in the network separately. The node representation learning process consists of a local GNN and a global GNN. The local consistency implementation of the graph is to directly use the initial adjacency matrix to perform the graph convolution^16^ operation. This adjacency matrix can be the default or otherwise constructed as a single graph. The implementation method of the global consistency of the graph is the graph convolution operation based on random walk processing introduced in the previous section. This is shown below.

1. $\mathrm{ConvA}$ (i.e., local consistency network). It adopts the GCN model proposed by Kipf^16^. We briefly describe $ConvA$ as a deep feedforward neural network. Input a feature set X and an adjacency matrix A, and output the embedding Z of the i-th hidden layer of the network as:
  6 $$\begin{array}{c}{Conv}_{A}^{\left(i\right)}\left(X\right)={Z}^{\left(i\right)}=\sigma \left({\widetilde{D}}^{-\frac{1}{2}}\widetilde{A} {\widetilde{D}}^\frac{1}{2}{Z}^{\left(i-1\right)}{W}^{\left(i\right)}\right)\end{array}$$
  where $\widetilde{A}=A+{I}_{n}$ is the adjacency matrix with self-loop (${I}_{n}\in {\mathbb{R}}^{N\times N}$ is the unit matrix) and ${\widetilde{D}}_{i,i}=\sum_{j}{\widetilde{A}}_{i,j}$. Therefore, ${\widetilde{D}}^{-\frac{1}{2}}\widetilde{A} {\widetilde{D}}^\frac{1}{2}$ is the normalized adjacency matrix. ${Z}^{\left(i-1\right)}$ is the output of the (I − 1)th level, ${Z}^{\left(0\right)}=X$. ${W}^{(i)}$ is the trainable parameter of the network, and $\upsigma \left(\cdot \right)$ denotes the activation function.
2. $\mathrm{ConvP}$ (i.e., global consistency network). Here, we introduce the convolutional approach of PMI to encode global information in another GNN network. It extracts more comprehensive semantic information, represented as a matrix $P\in {\mathbb{R}}^{N\times N}$. $ConvP$ is derived from the similarity defined by the PMI matrix, indicating that graph nodes appearing in similar contexts tend to have the same label. This neural network is given by the following equation:
  7 $$\begin{array}{c}{Conv}_{P}^{\left(i\right)}\left(X\right)={Z}^{\left(i\right)}=\sigma \left({D}^{-\frac{1}{2}}P{D}^\frac{1}{2} {Z}^{\left(i-1\right)}{W}^{\left(i\right)}\right)\end{array}$$
  where P is the PMI matrix and ${D}_{i,i}=\sum_{j}{P}_{i,j}$ is used for normalization. Global consistency is ensured by using diffusion based on such a node context matrix P. Moreover, $ConvP$ uses a similar neural network structure as $ConvA$. It has a higher degree of model coupling, which is convenient for model sharing and parameter transfer. With shared parameters, a node embedding module learns the representation using input from the source and destination domains.

### Attention mechanism module

For each domain, since the embeddings from the local and global consistency networks differ, i.e., contribute differently to the representation of graph learning, we use the graph attention module to obtain the importance of each domain's output. After performing graph convolution to extract features for the source and target domains, we obtain 4 embeddings from the local or global source graph embeddings ${Z}_{A}^{S}$, ${Z}_{P}^{S}$, and the local or global target graph embeddings ${Z}_{A}^{T}$, ${Z}_{P}^{T}$. The next step is to combine the aforementioned 4 embeddings from various graphs to create a single representation.

We use the raw inputs ${X}^{S}$ and ${X}^{T}$ as the keys to the attention mechanism. Then, we perform the attention mechanism on each of the above domain outputs, resulting in two attention coefficients, ${att}_{A}^{K}$ and ${att}_{A}^{K}$. This is calculated from the attention function $f\left(\cdot \right)$ for each domain as follows.

8 $$\begin{array}{c}{att}_{A}^{K}=f\left({Z}_{A}^{K},J{X}^{K}\right)\end{array}$$

9 $$\begin{array}{c}{att}_{P}^{K}=f\left({Z}_{P}^{K},J{X}^{K}\right)\end{array}$$

10 $$\begin{array}{c}{Z}^{S}={att}_{A}^{S}\cdot {Z}_{A}^{S}+{att}_{P}^{S}\cdot {Z}_{P}^{S}\end{array}$$

11 $$\begin{array}{c}{Z}^{T}={att}_{A}^{T}\cdot {Z}_{A}^{T}+{att}_{P}^{T}\cdot {Z}_{P}^{T}\end{array}$$

where $J$ is the shared weight matrix, and $K$ indicates whether the output is from the source domain S or the target domain T. This ensures that the input X has the same dimension as the outputs ${Z}_{A}^{K}$ and ${Z}_{P}^{K}$. Next, we use the $softmax$ function to normalize the weights ${att}_{A}^{K}$ and ${att}_{P}^{K}$. After performing the attention mechanism, we can get the final outputs ${Z}^{S}$ and ${Z}^{S}$.

### Domain adaptive module for cross-domain node classification

We propose a model made up of a source classifier, a domain adversarial module, and a target classifier that jointly learn class discrimination and domain invariant node representation to improve the classification of nodes in the target network. This model aims to improve knowledge acquisition across domains and knowledge transfer to help with the task of node classification across domains. The model's loss function is constructed as follows:

12 $$\begin{array}{c}L\left({Z}^{S},{Y}^{S},{Z}^{T},{Y}^{T}\right)={\mathcal{L}}_{S}\left({Z}^{S},{Y}^{S}\right)+{\gamma }_{1}{\mathcal{L}}_{DA}\left({Z}^{S},{Z}^{T}\right)+{\mathcal{L}}_{T}\left({Z}^{T},{Y}^{T}\right)\end{array}$$

${\mathcal{L}}_{S}$, ${\mathcal{L}}_{DA}$, and ${\mathcal{L}}_{T}$ stand for the source classifier loss, domain adversarial loss, and target classifier loss, respectively. ${\gamma }_{1}$ is the balance parameter.

1. **Loss of the source classifier.** The source classifier loss ${\mathcal{L}}_{S}\left({f}_{s}\left({Z}^{S}\right),{Y}^{S}\right)$ is to minimize the cross-entropy loss between the labeled data and the true labels in the source domain, as defined by Eq. (13).
  13 $$\begin{array}{c}{\mathcal{L}}_{S}\left({f}_{s}\left({Z}^{S}\right),{Y}^{S}\right)=-\frac{1}{{N}_{S}}\sum_{i=1}^{{N}_{S}}{Y}_{i}\mathrm{log}\left(\widehat{{Y}_{i}}\right)\end{array}$$
  where ${Y}_{i}$ denotes the true label of the ith node in the source domain, and $\widehat{{Y}_{i}}$ denotes the predicted class of the i-th source-labeled node ${V}_{i}^{S}$.
2. **Loss of the adversarial domain.** The goal of the domain adversarial loss ${\mathcal{L}}_{DA}\left({Z}^{S},{Z}^{T}\right)$ is to minimize the difference in node representation between the source domain network Gs and the target domain network Gt extracted by the convolutional layer to ensure that the model can perform parameter transfer and adversarial learning on two different domains. To achieve this, we add the domain classifier ${f}_{d}\left({Q}_{\lambda }\left({Z}^{S},{Z}^{T}\right);{\theta }_{D}\right)$ with the learning parameter ${\theta }_{D}$, which uses adversarial training to try to distinguish whether the nodes are from Gt or Gs. On the one hand, we want the source classifier ${f}_{s}$ to minimize Eq. (15) during the transfer process and correctly classify each node in the source domain. On the other hand, we want nodes from various domains to have representations that are as similar as possible, making it impossible for the domain classifier to tell if a node is from Gs or Gt (i.e., generative adversarial ideas).

Adversarial-based deep transfer learning is inspired by generative adversarial networks (GANs)^37–40^, which possess good learning and scalability. In our article, we implement adversarial training using the gradient reversal layer (GRL)^27^. GRL is defined in Eqs. (14) and (15) as ${Q}_{\lambda }\left(x\right)$ with inverted gradients. In contrast, the learning process of GRL is adversarial, forcing ${f}_{s}\left({Z}^{S}\right)$ to be maximized by inverting the gradient and optimizing ${\theta }_{D}$ by minimizing the loss of the cross-entropy domain classifier.

14 $$\begin{array}{c}{Q}_{\lambda }\left(x\right)=x\end{array}$$

15 $$\begin{array}{c}\frac{{\partial Q}_{\lambda }\left(x\right)}{\partial x}=-\lambda I\end{array}$$

16 $$\begin{array}{c}{\mathcal{L}}_{DA}=-\frac{1}{{N}^{S}+{N}^{T}}\sum_{i=1}^{{N}^{S}+{N}^{T}}{m}_{i}\mathrm{log}\left({\widehat{m}}_{i}\right)+\left(1-{m}_{i}\right)\mathrm{log}\left(1-{\widehat{m}}_{i}\right)\end{array}$$

where ${\widehat{m}}_{i}$ denotes the domain prediction of the i-th document in the source and target domains and ${m}_{i}\in \left\{\mathrm{0,1}\right\}$ signifies the base fact.

3. **Loss of target classifier.** In the target domain, a specific entropy loss is placed on the target classifier. Consistent with the source classifier, we use cross-entropy as the label loss for supervised learning in the target domain.
  17 $$\begin{array}{c}{\mathcal{L}}_{S}\left({f}_{t}\left({Z}^{T}\right),{Y}^{T}\right)=-\frac{1}{{N}_{T}}\sum_{i=1}^{{N}_{T}}{Y}_{i}\mathrm{log}\left(\widehat{{Y}_{i}}\right)\end{array}$$
  where ${Y}_{i}$ denotes the true label of the i-th node in the target domain, and $\widehat{{Y}_{i}}$ denotes the predicted class of the labeled node ${V}_{i}^{S}$ in the i-th target domain. The parameters in ${\mathcal{L}}_{S}\left({Z}^{S},{Y}^{S}\right)$, ${\mathcal{L}}_{DA}\left({Z}^{S},{Z}^{T}\right)$, and ${\mathcal{L}}_{T}\left({Z}^{T},{Y}^{T}\right)$ are jointly optimized by the objective function in Eq. (12). All parameters are optimized using the standard back-propagation algorithm.

## Conclusions

In this paper, we study the problem of cross-domain GNN transfer learning in healthcare. Most existing GNN learn models only in a single graph and do not consider knowledge transfer across graphs. Therefore, we propose a novel adversarial domain-adapted multichannel graph convolutional network (DAMGCN) to enable knowledge transfer between different CHD datasets. By using a dual graph convolutional network to aggregate the local and global relationships of graphs, we are able to learn better node representations in both source and target graphs. And the attention mechanism is combined to further generate uniform embeddings for downstream node classification tasks. By using cross-entropy loss in source domain classification, domain adversarial loss in domain differentiation, and cross-entropy loss in target domain information classification, we are able to reduce domain differences and achieve effective domain adaptation. We conducted experiments on three real CHD datasets, and the results show that our model outperforms existing single-domain network node classification methods and is able to distinguish well between high-risk CHD and low-risk CHD patient groups. This excellent predictive performance provides an aid to physicians in the diagnosis of cardiovascular problems.

## Data Availability

All data generated or analyzed during this study are included in this manuscript. The source code of the model and the datasets can be accessed from “https://github.com/xueyutao/Domain-adaptive-Transfer-Learning-with-Graph-Convolutional-Networks”.
